# Forecasting and control of emerging infectious forest disease through participatory modelling

**DOI:** 10.1098/rstb.2018.0283

**Published:** 2019-05-20

**Authors:** Devon A. Gaydos, Anna Petrasova, Richard C. Cobb, Ross K. Meentemeyer

**Affiliations:** 1 Department of Forestry and Environmental Resources, North Carolina State University, 2800 Faucette Drive, Raleigh, NC 27606, USA; 2 Center for Geospatial Analytics, North Carolina State University, 2800 Faucette Drive, Raleigh, NC 27606, USA; 3 Department of Natural Resources and Environmental Science, California Polytechnic State University, San Luis Obispo, CA 93407, USA

**Keywords:** participatory research, stakeholder engagement, landscape epidemiology, forest disease, geospatial, tangible interaction

## Abstract

Epidemiological models are powerful tools for evaluating scenarios and visualizing patterns of disease spread, especially when comparing intervention strategies. However, the technical skill required to synthesize and operate computational models frequently renders them beyond the command of the stakeholders who are most impacted by the results. Participatory modelling (PM) strives to restructure the power relationship between modellers and the stakeholders who rely on model insights by involving these stakeholders directly in model development and application; yet, a systematic literature review indicates little adoption of these techniques in epidemiology, especially plant epidemiology. We investigate the potential for PM to integrate stakeholder and researcher knowledge, using *Phytophthora ramorum* and the resulting sudden oak death disease as a case study. Recent introduction of a novel strain (European 1 or EU1) in southwestern Oregon has prompted significant concern and presents an opportunity for coordinated management to minimize regional pathogen impacts. Using a PM framework, we worked with local stakeholders to develop an interactive forecasting tool for evaluating landscape-scale control strategies. We find that model co-development has great potential to empower stakeholders in the design, development and application of epidemiological models for disease control.

This article is part of the theme issue ‘Modelling infectious disease outbreaks in humans, animals and plants: epidemic forecasting and control’. This theme issue is linked with the earlier issue ‘Modelling infectious disease outbreaks in humans, animals and plants: approaches and important themes’.

## 1. Introduction

On the 100th year anniversary of the Spanish flu pandemic, there is a need to reflect on how well epidemiological models, a fundamental tool of disease research, meet the needs of stakeholders involved with the day-to-day control of emerging outbreaks. With 100 years of history for perspective, it is clear that understanding the pattern and rate of spread is fundamental for designing successful policies and interventions [1–7]. Epidemiological models are powerful tools for evaluating disease spread under a range of possible conditions, and are especially useful for comparing intervention strategies, as experimental studies may be unethical, impractical or impossible [6–10]. However, the technical skill required to synthesize and operate these tools can restrict their use by the stakeholders who have the most to gain from them [10,11].

Several important examples highlight the value of models in guiding policy, especially regarding human diseases where forecast models have received great attention [12–14]. However, stakeholders rarely interact with such models directly, fuelling the argument that models are underused by stakeholders [6,7,10,11,15–17]. Reasons commonly cited for this knowledge–practice gap include: (i) models do not address stakeholder concerns, (ii) outputs cannot be clearly translated into policy or intervention, (iii) processes are explained poorly and/or are too esoteric, (iv) assumptions are considered invalid, and (v) models lack intuitive interfaces that facilitate stakeholder use [7,10,11,15–18]. These factors are compounded by the fact that models are often developed without systematic and transparent stakeholder input [11,15–17]. Essentially, modelling becomes a top–down exercise driven by the developers, with limited opportunities for stakeholders to guide model development, structure simulations or apply counterfactual analysis. This presents a dilemma for the field of epidemiological modelling, as models without stakeholder confidence will be ineffective at addressing the environmental, economic and social implications of disease [7,10,11].

Participatory research seeks to overcome this knowledge–practice gap by involving stakeholders who could be affected by research outcomes [15–17,19–22]. Owing to the rise of participatory approaches, there is an increased appreciation of the value of local knowledge, and an understanding that stakeholders and researchers have much to learn from each other [16,17,19–23]. Within the broader context of participatory research, we focus on two subdisciplines related to epidemiological modelling: participatory modelling (PM) and participatory epidemiology (PE). Originally developed to improve research and control of livestock diseases in data-scarce regions, PE uses techniques like semi-structured interviews, participatory mapping and participatory disease surveillance to acquire fundamental data and situate epidemiological research in local contexts [22,24–26]. Importantly, PE may or may not include models. By contrast, PM arose to support decision-making in natural resource management where conflicting stakeholder interests play an integral role in management success [15–17]. By definition, PM involves stakeholders in modelling, but the types of models used can be variable, ranging from conceptual models to more complex geospatial simulation models [15–17]. Despite differences in focus, PE and PM share similar techniques and motivations for involving stakeholders. Further, when PE applications feature models, they fall within the sphere of PM.

Here, we demonstrate through a systematic literature review and an ongoing case study of sudden oak death (SOD) that PM approaches are currently rare in studies of plant disease modelling, but have great value for collaboratively exploring control strategies. Since its introduction, *Phytophthora ramorum*, the cause of SOD, has precipitated significant ecological and economic damage along the Pacific coast of the United States, with impacts including loss of foundational tree species, increased wildfire hazards and increased costs associated with plant trade [9,27–31]. Southwestern Oregon has recently experienced a resurgence of public concern because of a second introduction event, the potentially more aggressive European 1 (EU1) lineage [32,33]. Coordinated management to curb disease spread could reduce economic consequences from the quarantine of nursery and forestry products in surrounding counties [9,31,34]. Both in our example, and in epidemiology broadly, modelling tools can help stakeholders address critical questions regarding where management will be most effective, when eradication becomes implausible, and if the pathogen is likely to escape quarantine. Using a PM approach, we worked with local stakeholders to collaboratively develop an interactive modelling tool for analysing *P. ramorum* management in Oregon. Here, we focus on how stakeholder involvement has shaped our research goals, the functionality and parametrization of the epidemiological simulation and the development of Tangible Landscape, an interactive technology for decision-making (see: https://tangible-landscape.github.io/) [18,35]. This case study of model co-development illustrates the potential benefits of PM to empower stakeholders in the design, development and application of epidemiological models for disease control.

## 2. Methods

### (a) Literature review

PM and PE share similar motivations for engaging stakeholders [16,17,22,24–26], but it is unclear how much overlap exists between these fields. To understand the extent and manner in which PM is currently employed within epidemiology, we conducted two systematic literature analyses using the ISI Web of Science Database. One search focused on areas of overlap between PE and PM across all disease systems (human, animal and plant), with the other search focusing specifically on instances of stakeholder participation in plant epidemiology. Sources were first evaluated based on the modelling framework. Any sources that included models of disease spread or risk were assessed in-depth and categorized based on disease system (human, animal or plant) and type of stakeholder participation. These searches can be reproduced with details included in electronic supplementary material, Appendix SA.

Engagement exists on a spectrum with varying degrees of stakeholder control [16,20,36,37]; therefore, we divided sources into three broad categories of participation: (i) only mentioned, where there was no participation, but it was mentioned that stakeholders would benefit the research or benefit from the research; (ii) contributed data, where stakeholders provided disease locations, parameter estimates or information about their actions; and (iii) co-development, where stakeholder input significantly affected development of the model, scenarios or interface, often in an iterative manner. All levels of engagement can be valid and productive; however, participatory research ultimately strives for co-development where stakeholders increasingly steer research goals, processes and outcomes [16,17,19,20,37].

### (b) Sudden oak death reemergence

Consultation with stakeholders early in research development identified the potential for collaborative planning to improve *P. ramorum* management in southwestern Oregon. Although *P. ramorum* had been present in this region since 2001 [31,38], public concern had been reignited following the introduction and laboratory confirmation of the EU1 lineage [32,33], which is more virulent than the previously established North American (NA1) lineage [39]. There is also evidence that EU1 can infect Douglas fir (*Pseudotsuga menziesii*) and grand fir (*Abies grandis*) seedlings, potentially placing critically important regional timber products at risk [33]. Without a concerted and coordinated intervention, there is justified concern that this new strain could quickly spread into surrounding counties, making this an ideal case for leveraging the power of predictive models to explore the implications of proposed responses.

### (c) Tangible landscape modelling tool

To simulate disease spread in Oregon, we adapted an existing modelling framework originally developed to explore disease dynamics in California [5,18,40]. These stochastic, spatially explicit simulations integrate the effects of host density and weather conditions on pathogen transmission and establishment. For a detailed model description, including key data requirements, model processes and links to source code, see electronic supplementary material, Appendix SB; [5,18,40].

Geospatial models can be perceived as inaccessible because their use requires substantial technological skill [7,16,17]. To overcome this barrier, the disease simulation was coupled with Tangible Landscape, a geospatial PM platform (see: https://tangible-landscape.github.io/). With Tangible Landscape, users guide the model intuitively through physical actions, rather than through code or software [18,35]. Stakeholders place markers on a visualization of the study area to designate the spatial allocation of host removal. These locations serve as input for the disease simulation by altering host density data. The resulting model outcomes are visualized on the study area, allowing users to quickly and intuitively assess how their actions affected disease spread [18,35]. A pilot study using Tangible Landscape to evaluate SOD control measures found that users quickly learned model processes, intuitively explored management scenarios and learned from other participants, making this system ideal for PM applications [18]. For further discussion of the Tangible Landscape tool, see electronic supplementary material, Appendix SB.

### (d) Participatory modelling workshops

Reciprocal feedback between model developers and stakeholders battling the EU1 infestation was engendered through a participatory workshop in October 2017. Workshop goals were to: (i) assess how participants interact with the model, and (ii) systematically collect stakeholder input to refine model dynamics and the Tangible Landscape interface. While PM strives to incorporate the full diversity of opinions, engaging stakeholders at all stages of model development may not be necessary or advisable as stakeholders often face significant time constraints or rely on developers for specific tasks [16,20,41]. In our case, there was an emphasis on refining the epidemiological model and we therefore concentrated on stakeholders with knowledge of local disease dynamics. Stakeholders with relevant expertise were invited from the US Forest Service, Oregon Department of Forestry and Oregon State University, and were encouraged to invite others who may be interested. We limited the maximum number of participants to 20, as previous experience suggests smaller groups allow for more personal interaction with the Tangible Landscape tool.

All participants were asked to complete questionnaires designed to assess participants' baseline knowledge, self-reported learning, interactions with Tangible Landscape, confidence in model processes and data, and recommended improvements. Because the aim of these surveys was to systematically collect suggestions for improvement, we strove for a broad spectrum of feedback. Questionnaires were developed following guidelines in [42,43], and included a mix of rank-choice, open-ended and Likert scale questions. Surveys were tested prior to the workshop by researchers not connected to the project. For further description, including participatory workshop outline and survey questions, see electronic supplementary material, Appendix SC.

## 3. Results

### (a) Literature review results

**Figure 1.**
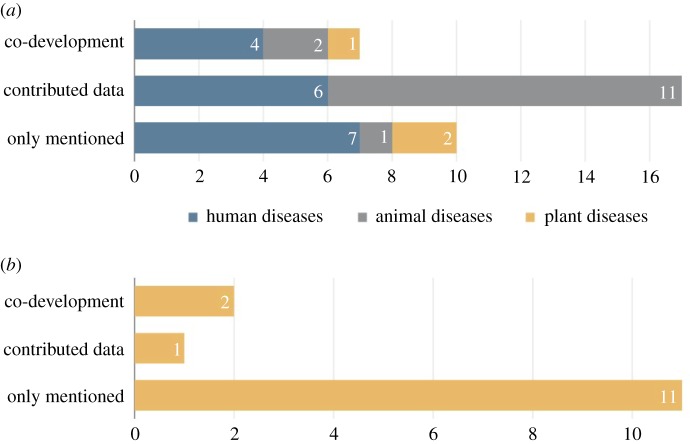
Summary of literature searches examining (*a*) the role of PM across disease systems, and (*b*) the role of stakeholder engagement in plant disease modelling. (Online version in colour.)

Our two literature searches examined the role of PM in disease forecasting and control across all systems (human, animal and plant) and stakeholder engagement in plant epidemiology in particular (figure 1). Only sources about modelling disease spread or potential were considered, resulting in 29 and 14 sources, respectively. For data presented in figure 1, sources could be counted more than once if they reported multiple case studies that fell under different disease or participation categories, for example [24]. A majority of studies focused on diseases of humans (17) and animals (14), with few touching on plant disease (3) (figure 1*a*). Among all participatory studies, the majority of interactions revolved around contributing data (17) with fewer instances of co-development (10). Notably, there was only one plant disease study in the co-development or contributed data categories. This pattern was further illustrated by our second literature search examining how stakeholders are being engaged specifically in modelling of plant diseases (figure 1*b*). Among these sources, the majority were classified in the lowest category of interaction ‘only mentioned’ (11), with fewer instances of contributing data (1) or co-development (2).

### (b) Workshop results

**Figure 2.**
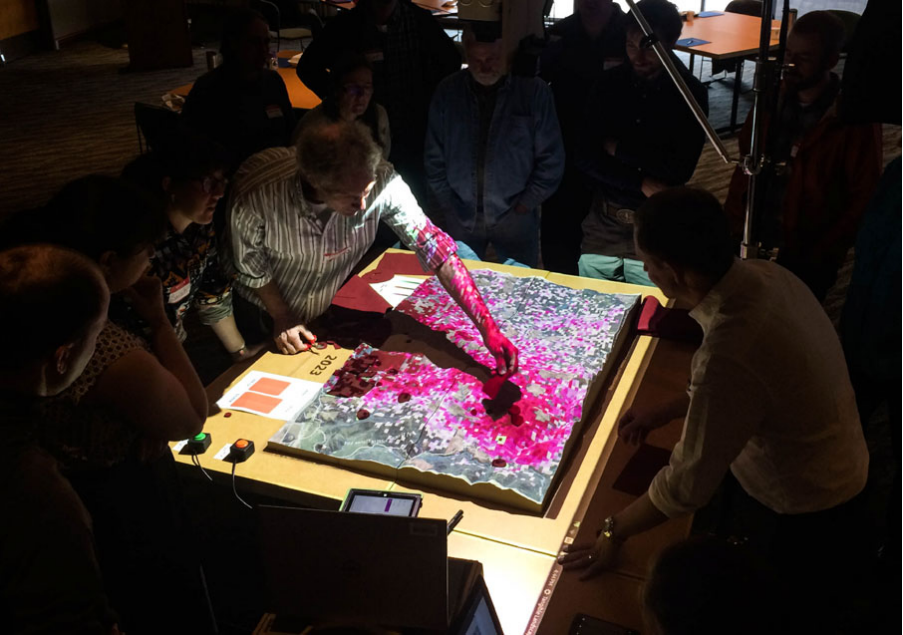
Participants interact with disease model at workshop. Credit: Rich Cobb. (Online version in colour.)

**Figure 3.**
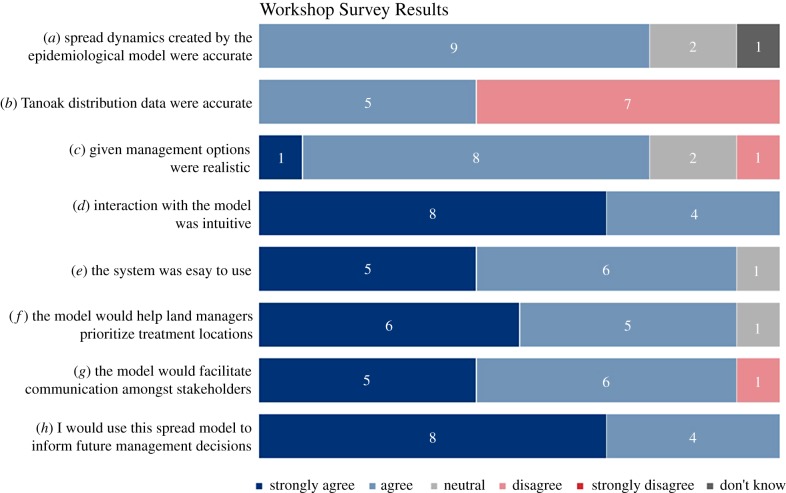
Survey responses evaluated core model components, including spread dynamics (*a*), host data (*b*), management options (*c*), interaction (*d*), accessibility (*e*), ability to prioritize treatment locations (*f*) and ability to facilitate communication (*g*). Participants also reported willingness to use the model (*h*). (Online version in colour.)

Twelve stakeholders from the US Forest Service, Oregon Department of Forestry and Oregon State University participated in a modelling workshop to perform management experiments with and provide feedback on an interactive tool for disease forecasting and control (figure 2). Although the focus on this group restricts the range of stakeholder opinions, the approach produced highly relevant feedback on epidemiological processes and management scenarios. Many of the participants indicated prior familiarity with disease forecasting models (7), although only 3 had used these tools in management planning (electronic supplementary material, Appendix SC). Survey results indicate that most participants found the modelling tool intuitive and easy to use (figure 3*d*,*e*), aiding the perception that the tool would be useful for prioritizing treatment locations and facilitating communication among stakeholders (figure 3*f*,*g*). Encouragingly, all stakeholders indicated that they learned something through the workshop, and that they would be likely to use the model to inform future management decisions (figure 3*h*, electronic supplementary material, Appendix SC). While many aspects of the system and workshop were rated highly, the most valuable results were those indicating areas where the model could be improved. Participants were sceptical of the resolution and accuracy of the underlying host distribution data and felt inadequacies here could influence the spatial dynamics of the simulation (figure 3*a*,*b*). Stakeholders provided guidance on ways to refine these aspects, among others, to make the tool more useful in management planning. Further details, including open-ended responses about self-assessed learning and suggested model changes, can be found in electronic supplementary material, Appendix SC.

## 4. Discussion

**Figure 4.**
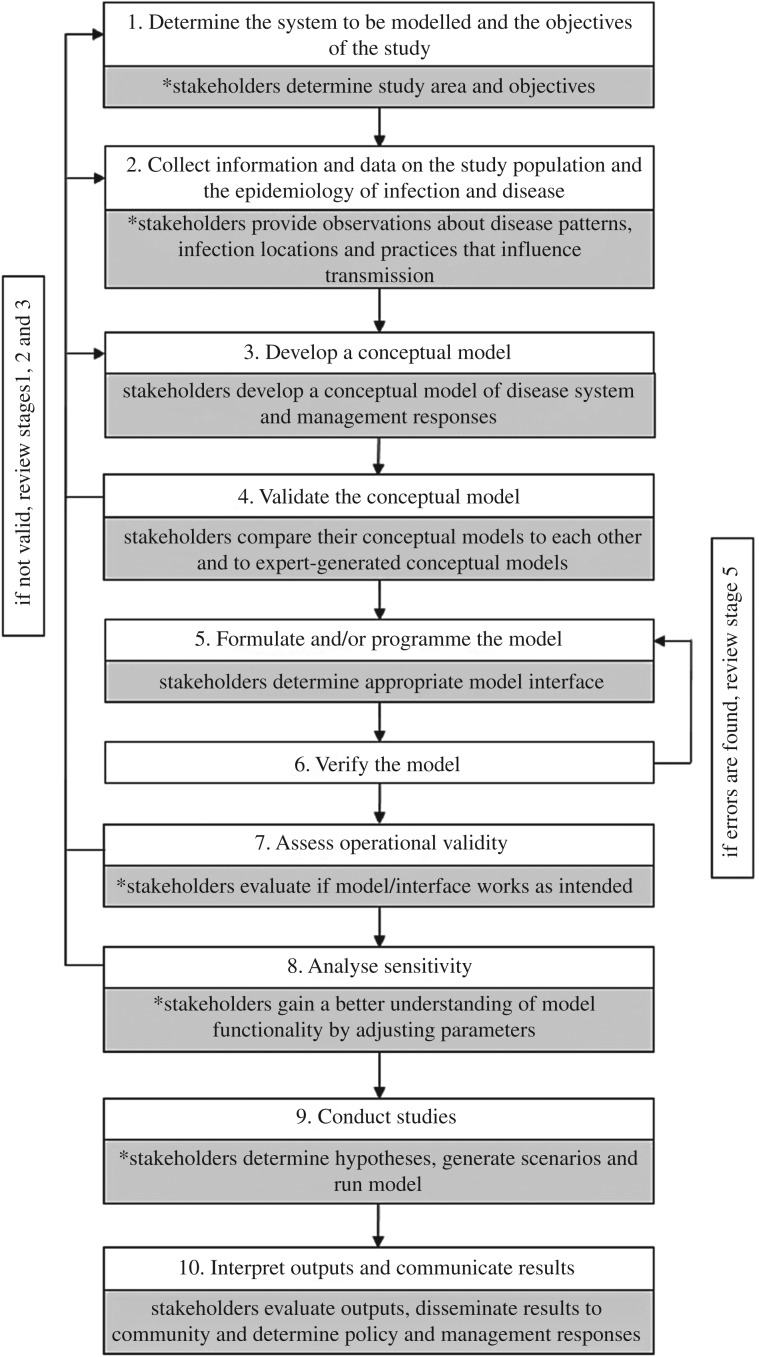
An adaptation of Garner & Hamilton's [1] framework of epidemiological model development. White boxes depict stages of model development [1], with grey boxes indicating how stakeholders can contribute to these stages. Asterisks highlight ways we have engaged stakeholders throughout this case study.

It is increasingly recognized that stakeholders possess local knowledge of disease transmission, risk factors and control strategies, and can be instrumental in guiding and implementing actionable epidemiological research [6,19,22–26]. PM, which integrates these diverse perspectives throughout model development, encourages collaborative learning and empowers stakeholders to interact more directly with models [15–17,24]. We adapted the framework of Garner & Hamilton [1] to demonstrate how stakeholders can contribute to each stage of epidemiological model development (figure 4). While this is not an exhaustive list of potential stakeholder contributions, each of these interactions has proven valuable in PE or PM research to date [16,17,22,24–26]. We engaged stakeholders at multiple stages of model development (figure 4) and this resulted in a deeper understanding of the study system by the developers while making our model more applicable for a specific application by the stakeholders. In our case study, the use of PM techniques is justified both in terms of model improvements and in increased usability and confidence from the stakeholders. More broadly for plant epidemiology, PM techniques are relatively underused and our example suggests that it is a potential approach for empowering stakeholders to apply model insights to address disease spread and impacts [6,23,44–48].

Constructing overarching guidelines for PM applications is a growing research topic [15–17,20,41], but several principles have emerged that are highlighted by our example. Models that are inaccessible to stakeholders are likely to be overlooked; therefore, intuitive interfaces are essential for PM [7,16–18,48]. Coupling the epidemiological model with Tangible Landscape encouraged participants to collaboratively design management scenarios, visualize underlying spatial data and assess the stochastic uncertainty of results [18,35]. In our surveys, participants overwhelmingly rated the system as intuitive and easy to use (figure 3*d*,*e*) and we expect this was integral to promoting communication. The visualization capabilities proved especially beneficial, with one participant adding, ‘[it] reaffirmed [my] belief that effective visual displays are helpful in learning. Even more effective is the self-learning gained by “gaming” the system. Would be extremely helpful with stakeholders' (electronic supplementary material, Appendix SC). A majority of participants agreed, indicating that the system would facilitate collaboration (figure 3*g*). Previous work with Tangible Landscape [18] demonstrated that the platform can help users quickly grasp and communicate essential model properties. For PM to be applied in other disease systems, assessing and improving the interface will likely be of critical importance.

Our workshop also highlighted a knowledge–practice gap that is likely common to many disease systems. Several participants indicated familiarity with disease models, but few had used them for management planning (electronic supplementary material, Appendix SC). Collaborative learning, a fundamental goal of the PM process, is theorized to help narrow this gap [15–17,41]. During the workshop, model exploration sparked discussions about disease dynamics, detection protocols, field operations and local concerns. One participant noted ‘many aspects of spread and treatment dynamics came out from the various participants. It was very interesting to have the breadth of knowledge in the room’ (electronic supplementary material, Appendix SC). All participants indicated they would be likely to use the spread model to inform future management decisions (figure 3*h*), highlighting that collaboration between stakeholders and researchers can elucidate the value of epidemiological models.

Collaborative learning fundamentally affects all parties, and we found that engaging stakeholders impacted our research in considerable ways. Early discussions led us to refocus our attention on southwestern Oregon, where there is a pressing need for disease forecasting and control. Consequently, we worked with stakeholders to adapt a pre-existing disease model to represent local transmission dynamics and to address the most urgent needs of decision-makers. While participants rated many aspects of the Tangible Landscape highly, survey results were mixed considering the accuracy of the underlying epidemiological model, specifically spread processes, host data and management options (figure 3*a*–*c*). Participants provided guidance on how to improve these areas, with suggestions including: (i) generating higher-resolution host maps that account for effects of prior timber harvest, (ii) modelling pathogen strains separately, (iii) adding management options, (iv) allowing yearly management interventions, and (v) allowing easy parameter variations (electronic supplementary material, Appendix SC). These changes to the model structure or outputs represent unique contributions of the participants that the developers would not have pursued otherwise. Our subsequent research efforts have focused directly on these model deficiencies, and we believe that integrating these suggestions will make the modelling tool more applicable for disease forecasting and control in Oregon. Following the incorporation of these improvements, we will hold additional workshops with a larger, more diverse group of Oregon stakeholders to seek further input.

PM is not the only way to increase collaboration between stakeholders and researchers, and whether or not to employ a participatory approach requires critical reflection and depends on the research and management goals. Model co-production can be expensive, time-consuming and requires significant commitment from stakeholders and researchers alike [15–17,19,41]. Even with the best intentions, participatory research can fall short of achieving actionable science [19]. For these reasons, PM is best applied to problems with diverse actors and significantly complex socio-ecological interdependencies [15–17,41]. The management of epidemics frequently fits this definition [2–3,6,9,10,22]. Within epidemiology, there are often substantial challenges associated with model or simulation complexity. Rigorous model development to assess validity is just as essential to PM as any other epidemiological model (figure 4) [1,24]. Stakeholder engagement enhanced our model development by integrating new and diverse perspectives; we expect that similar improvements in development efficiency and overall application can be made by employing the PM approach.

Our literature analysis supports the perspective that PM has not yet reached its full potential in epidemiology, especially in regard to plant disease modelling (figure 1). As both a driver and deterrent of plant disease spread, stakeholders often determine the success of control strategies [6,23,44–48]. Other researchers have argued for increased stakeholder engagement within plant disease modelling, but PM methodologies are only beginning to be explored [6,23,44–48]. In our case study of *P. ramorum*, incorporating a PM framework resulted in a co-developed model and signs that this approach is changing perspectives on the role of models in management and policy decisions. Understanding if model co-development actually changes behaviour, for example, by changing field decisions in the light of model results, remains to be seen. Regardless, stakeholder involvement clearly improved our modelling tool by guiding our research goals, model development, and by creating a forum for communication. This suggests that PM has potential to help bridge the knowledge–practice gap by facilitating collaborative learning and empowering stakeholders in the design, development and application of epidemiological models for plant disease control.

## Supplementary Material

Appendix A: Systematic Literature Review

## Supplementary Material

Appendix B: Epidemiological Modelling Tool

## Supplementary Material

Appendix C: Participatory Modelling Workshop

## Data accessibility

Tangible Landscape and the epidemiological spread model are available under GNU General Public License, and can be downloaded as described in electronic supplementary material, Appendix SB. All data for literature reviews are submitted along with this manuscript and are described under electronic supplementary material, Appendix SA. Relevant survey data can be found in electronic supplementary material, Appendix SC.
