# Supplementary material for: Forecasting and control of emerging infectious forest disease through participatory modelling
Source: Philos Trans R Soc Lond B Biol Sci. 2019 May 20;374(1776):20180283. doi: 10.1098/rstb.2018.0283 (PMC6558554; doi:10.1098/rstb.2018.0283)
Supplement: Appendix A: Systematic Literature Review [file rstb20180283supp1.docx]

Electronic Supplemental Material for “Forecasting and control of emerging infectious forest disease through participatory modelling”

Appendix A: Systematic Literature Review:

We conducted systematic literature reviews addressing two questions: 1) how is participatory modelling being applied in studies modelling disease spread or potential across all systems (plant, animal, and human)? and, 2) how are stakeholders being engaged specifically in studies of plant disease modelling? Searches were conducted in the ISI Web of Science database during the months of July and August 2018. We did not consider any sources that were not in English.

For our first question about participatory modelling in epidemiology, we compiled three searches using the following terms: 1) participatory model* epidemiolog*, 2) participatory model* with the infectious disease category selected, and 3) participatory epidemiolog* simulation model* *OR*  participatory disease simulation model* *OR*  participatory pathogen simulation model* *OR* participatory epidemiolog* spread model* *OR* participatory disease spread model* *OR p*articipatory pathogen spread model*. Some sources were found under multiple searches. In total, there were 103 sources. One source couldn’t be located, and one source was eliminated because it was not in English. For a source to be considered, it had to be about modelling of disease spread or potential. Twenty-nine sources fit this criteria and were considered more in-depth (Table A1).

For our second question about stakeholder engagement in plant disease modelling studies, we conducted one search with the terms: plant stakeholder* model* disease OR plant stakeholder* model* pathogen OR plant stakeholder* simulation disease OR plant stakeholder* simulation pathogen. In total, there were 43 sources. One source couldn’t be located, and one source was eliminated because it was not in English. For a source to be considered, it had to be about modelling of disease spread or potential. Fourteen sources fit this criteria and were considered more in-depth (Table A2).

We recognise important limitations to these kinds of literature reviews, most significantly the bias against publishing negative results. There may be an even greater disincentive to publish negative participatory modelling results, as ‘bad press’ can jeopardize management funding and erode essential interpersonal connections between stakeholders and modellers. Furthermore, by focusing specifically on models of disease spread or potential, we may have missed relevant participatory research with other types of models or in epidemiology more broadly. Lastly, it is possible that there are applicable developments reported in other languages or under different names.

| **Table A1: Participatory Modelling in Epidemiology Studies** | | | | |
| --- | --- | --- | --- | --- |
| **Source Title** | **First Author** | **Study Topic** | **Disease System** | **Participatory Category** |
| A heterogeneous population model for contagious bovine pleuropneumonia transmission and control in pastoral communities of East Africa | Mariner, J.C. | contagious bovine pleuropneumonia | animal | contributed data |
| A model of lineage-1 and lineage-2 rinderpest virus transmission in pastoral areas of East Africa | Mariner, J.C. | rinderpest virus | animal | contributed data |
| Moving alcohol prevention research forward Part 1: introducing a complex systems paradigm | Apostolopoulos, Y. | alcoholism | human | just mentioned |
| Moving alcohol prevention research forward Part 2: new directions grounded in community-based systems dynamics modeling | Apostolopoulos, Y. | alcoholism | human | just mentioned |
| How to reach the poor? Surveillance in low-income countries, lessons from experiences in Cambodia and Madagascar | Goutard, F.L. | various zoonotics | human and animal | contributed data |
| Impacts of climate change on plant diseases- opinions and trends | Pautasso, Marco | various plant diseases | plant | just mentioned |
| A model of contagious bovine pleuropneumonia transmission dynamics in East Africa | Mariner, J.C. | contagious bovine pleuropneumonia | animal | contributed data |
| Web-based participatory surveillance of infectious diseases: the Influenzanet participatory surveillance experience | Paolotti, D. | flu | human | contributed data |
| Application of system dynamics and participatory spatial group model building in animal health: A case study of East Coast Fever interventions in Lundazi and Monze districts of Zambia | Mumba, C. | East Coast fever | animal | co-development |
| Tangible geospatial modeling for collaborative solutions to invasive species management | Tonini, F. | sudden oak death | plant | just mentioned |
| Moving interdisciplinary science forward: integrating participatory modelling with mathematical modelling of zoonotic disease in Africa | Grant, C. | various zoonotic diseases including henipavirus, Lassa fever, Rift Valley fever, and trypanosomiasis | human and animal | contributed data and co-development |
| The economic and poverty impacts of animal diseases in developing countries: new roles, new demands of economics and epidemiology | Rich, K.M. | Rift Valley fever, avian influenza, and food and mouth disease | animal | just mentioned |
| An analytics framework to support surge capacity for planning emerging epidemics | Curran, M. | epidemics in general | human | just mentioned |
| Metapopulation dynamics and determinants of H5N1 highly pathogenic avian influenza outbreaks in Indonesian poultry | Farnsworth, M.L. | H5N1 avian influenza | animal | contributed data |
| Description and analysis of the poultry trading network in the Lake Alaotra region, Madagascar: implications for the surveillance and control of Newcastle disease | Rasamoelina-Andriamanivo, H. | Newcastle disease | animal | contributed data |
| Situated knowledge of pathogenic landscapes in Ghana: understanding the emergence of Buruli ulcer through qualitative analysis | Tschakert, P. | Buruli ulcer | human | co-development |
| Evaluating the efficiency of participatory epidemiology to estimate the incidence and impacts of foot-and-mouth disease among livestock owners in Cambodia | Bellet, C. | foot and mouth disease | animal | contributed data |
| Uncertainty in epidemiology and health risk and impact assessment | Briggs, P.J. | uncertainty in epidemiology | human | just mentioned |
| Identifying risk factors of highly pathogenic avian influenza (H5N1 subtype) in Indonesia | Leo, L. | avian influenza | animal | contributed data |
| A participatory approach to design spatial scenarios of cropping systems and assess their effects on phoma stem canker management at a regional scale | Hossard, L. | phoma stem canker | plant | co-development |
| Dynamic simulation modelling of policy responses to reduce alcohol-related harms: rationale and procedure for a participatory approach | Atkinson, J.A. | alcohol-related diseases | human | co-development |
| A blockchain-enabled participatory decision support framework | Laskowski, M. | disease in general | human | just mentioned |
| A participatory simulation model for studying attitudes to infection risk | Maharaj, S. | attitudes towards disease risk | human | just mentioned |
| A participatory model of the paradox of primary care | Homa, L. | various illnesses | human | co-development |
| Avian Cholera emergency in Arctic-nesting northern Common Eiders: using community-based, participatory surveillance to delineate disease outbreak patterns and predict transmission risk | Iverson, S.A. | avian cholera | animal | contributed data |
| Expert knowledge sourcing for public health surveillance: national tsetse mapping in Uganda | Berrang-Ford, L. | Human African typanosomiasis | human | contributed data |
| Combining public participatory surveillance and occupancy modelling to predict the distributional response of Ixodes scapularis to climate change | Lieske, D.J. | mapping tick prevalence | human and animal | contributed data |
| Spontaneous social distancing in response to a simulated epidemic: a virtual experiment | Kleczkowski, A. | social distancing in response to infection | human | contributed data |

Table A1. Sources considered for first search examining overlap between participatory modelling and participatory epidemiology.

| **Table A2: Stakeholder Engagement in Plant Disease Modelling Studies** | | | |
| --- | --- | --- | --- |
| **Source Title** | **First Author** | **Study Topic** | **Participatory Category** |
| When prevention fails. Toward more efficient strategies for plant disease eradication. | Vicent, A. | citrus canker | just mentioned |
| Risk-based management of invading plant disease | Hyatt-Twynam, S.R. | citrus canker | just mentioned |
| Monitoring invasive pathogens in plant nurseries for early-detection and to minimise the probability of escape | Chavez, A. | sudden oak death, HLB, citrus canker, ash dieback | just mentioned |
| A set of software components of the simulation plant airborne diseases | Bregaglio, S. | wheat brown rust and rice blast epidemics | just mentioned |
| Citizen science helps predict risk of emerging infectious disease | Meentemeyer, R.K. | sudden oak death | contributed data |
| Optimising and communicating options for the control of invasive plant disease when there is epidemiological uncertainty | Cunniffe, N.J. | citrus canker | just mentioned |
| Addressing the implementation problem in agricultural decision support systems: the example of vite.net (R) | Rossi, V. | viticultural diseases | co-development |
| Impacts of climate change on plant diseases - opinions and trends | Pautasso, M. | various plant diseases | just mentioned |
| Potential and limitations of plant virus epidemiology: lessons from the potato virus Y pathosystem | Doring, T.F. | potato virus Y | just mentioned |
| Tracking the distribution and impacts of diseases with biological records and distribution modelling | Purse, B.V. | various plant, animal, and human diseases | just mentioned |
| Epidemiology and population biology of Pseudoperonospora cubensis: a model system for management of downy mildews | Ojambo, P.S. | cucurbit downy mildew | co-development |
| Predicting the benefits of banana bunchy top virus exclusion from commercial plantations in Australia | Cook, D.C. | banana bunchy top virus | just mentioned |
| Plant pathogens, insect pests and weeds in a changing global climate: a review of approaches, challenges, research gaps, key studies and concepts | Juroszek, P. | various plant diseases | just mentioned |
| Clubroot of cruciferous crops - new perspectives of an old disease | Howard, R.J. | clubroot disease of brassicas | just mentioned |

Table A2. Sources considered for second search examining stakeholder engagement in plant disease modelling.
