# Supplementary material for: Forecasting and control of emerging infectious forest disease through participatory modelling
Source: Philos Trans R Soc Lond B Biol Sci. 2019 May 20;374(1776):20180283. doi: 10.1098/rstb.2018.0283 (PMC6558554; doi:10.1098/rstb.2018.0283)
Supplement: Appendix B: Epidemiological Modelling Tool [file rstb20180283supp2.docx]

Electronic Supplemental Material for “Forecasting and control of emerging infectious forest disease through participatory modelling”

Appendix B: Epidemiological Modelling Tool:

Disease Spread Model:

To model disease spread in Oregon, we expanded upon a pre-existing modelling framework originally developed to simulate the spread of *P. ramorum* in California [1-3]. While most epidemiological processes are the same in these two regions, key differences exist. In California, disease spread is predominantly influenced by two high-competency host trees: California bay laurel (*Umbellularia californica*) and tanoak (*Notholithocarpus densiflorus*). For unknown reasons, California bay laurel is found to be a low-competency host in Oregon which does not contribute significantly to disease spread [4-6]. Therefore, we amended the original multi-host framework to reflect a single-host system driven by tanoak. Evidence also suggests that *P. ramorum* is able to produce spores year-round in Oregon, likely due to wetter climatic conditions. Therefore, we removed the seasonality component which prevented spread during winter months in the California models.

The intensive management in Oregon presents a challenge for model parameterization and validation because it obscures natural patterns of disease transmission [5]. Due to this limitation, the model was parameterized based on natural spread conditions in Northern California following the Markov chain Monte Carlo (MCMC) process described in [2]. While there are some differences in epidemiology, this area is a tanoak-heavy ecosystem and we felt it provided a reasonable approximation for this case study. Prior to the workshop, we performed a qualitative analysis as described in [2], where we compared model outputs to: 1) known infection locations in Oregon, and 2) to a map of *P. ramorum* risk [7]. We found there was good visual correspondence between model outputs, the risk map, and known infection locations, and felt this was suitable for this stage of model development. We are currently pursuing more intensive quantitative validations which take into account the Oregon’s extensive treatments.

*Data Requirements*

This geospatial model requires raster inputs of host density, weekly weather conditions, and initial infection locations. Host density data for the main host, tanoak, was derived from detailed raster structure maps from the Landscape Ecology, Modeling, Mapping and Analysis (LEMMA) [8] project webpage (<https://lemma.forestry.oregonstate.edu/>) using the density calculation presented in [1]. Daily values of precipitation, minimum temperature and maximum temperature were obtained as raster data from the PRISM Climate Group [9] webpage (<http://www.prism.oregonstate.edu/>). Minimum and maximum temperature were converted to a measure of average temperature. These data were aggregated to a weekly timestep, and converted to an index of weather suitability for disease spread based on laboratory studies as reported in [1-2]. Infection locations were acquired from the Oregon Department of Forestry’s annual survey program for 2001 through 2017. These point locations were converted to raster data representing number of infected host trees per pixel.

*Model Processes*

The model is a spatially-explicit susceptible-infected (SI) model consisting of three stochastic processes: sporulation, spore dispersal, and spore establishment. The amount of spores produced by an infected host is sampled each week from a Poisson distribution as described in [2]. This spore-rate corresponds to the maximum number of new infections that could be produced from an infectious host, and is moderated by the weather conditions during that week.

Spore dispersal is controlled by a particle-emission anisotropic process as described in [1]. The direction of dispersal is sampled from a Von Mises circular probability distribution. The predominant wind direction for this study area is north, which was used to parameterize the angular distribution for the Von Mises distribution. The dispersal distance was sampled from a Cauchy probability distribution parameterized with values from [2]. Given the comparatively small study area, we did not include the long-distance component of the dispersal kernel (which accounts for human-mediated spread on a regional scale).

Spore establishment is governed by a stochastic process which probabilistically challenges the cell based on the amount of susceptible hosts and weekly weather conditions as described in [1]. If the value of hosts and weather suitability is higher than a randomly sampled number, one new infection will occur in that pixel. Infections can occur both in new cells, and within a cell that the spore originated.

*Case Study*

The disease simulation was run in a 12x8 km area surrounding Gold Beach, Oregon, where the recent European-1 (EU1) strain locations were found. Model resolution was 100m. The model was run at a weekly timestep for a period of 5 years, from 2016 to 2021, and from 2017 to 2022.

*Source Code*

This sudden oak death disease spread model is available as a GRASS GIS add-on module [10] (see user documentation: <https://grass.osgeo.org/grass74/manuals/addons/r.spread.sod.html>).

Tangible Landscape System:

*Description of Interface*

Tangible Landscape is a tangible user interface (TUI) which allows users to intuitively guide a geospatial model through a series of physical actions [11]. The system consists of a scanner, a projector, a computer with GRASS GIS software, a physical landscape terrain model, and USB-enabled buttons (Figs. B1 and B2). Tangible Landscape can be used for modelling many geospatial processes, but here we are using a customized set up specifically for pest and pathogen modelling [11]. Geospatial data layers, including aerial imagery, roads, property boundaries, infection locations, and host data are projected onto the physical landscape model to provide context. Users place felt markers on the landscape to represent management (e.g., culling hosts). These markers are detected by a scanner and converted to GIS polygons, which are then used to alter the host data used in the disease spread model. Disease spread is calculated using this new host input, and spread locations are projected back into the terrain model to give users a sense of how the proposed management scenario alters landscape-scale disease spread. Using the two USB-enabled buttons, users can switch back and forth between an animation of a single stochastic model iteration and a probability surface showing likelihood of infection within 10 model runs, giving users both a sense of how the disease may progress across the landscape and the stochastic uncertainty of that outcome.

*Dashboards*

Tangible Landscape is linked with two interactive dashboards to aid in designing and comparing management strategies. As users designate management locations, information about the area and cost of management is displayed next to the physical landscape to help guide scenario development (see steering dashboard, Fig. B2). Data from each scenario are stored in a web-based dashboard that allows users to compare dozens of scenarios simultaneously (see summary dashboard, Figs. B2 and B3). Scenarios are tracked by the name of the user who designated management locations, allowing people to track their individual model runs and compare their results to others’. Metrics in the web-based dashboard include number of infected trees, number of infected hectares, money spent, area treated, and price per protected tree.

*Equipment and Code*

All equipment and code for Tangible Landscape is open-source and runs with GRASS GIS platform [10]. For more information about Tangible Landscape, see <https://tangible-landscape.github.io/> or Tangible Modeling with Open Source GIS: Second Edition [11].


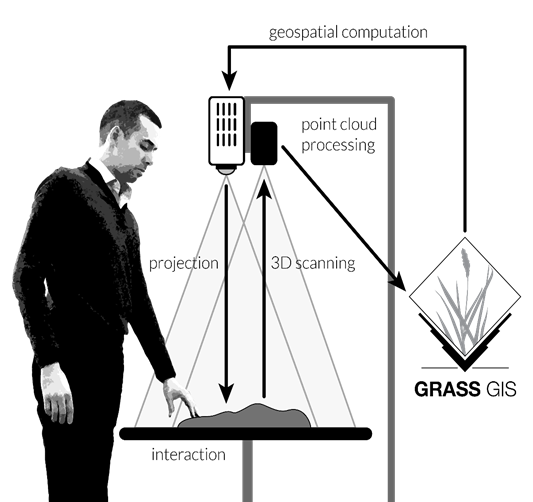


Figure B1. Schema of Tangible Landscape design. Credit: Brendan Harmon


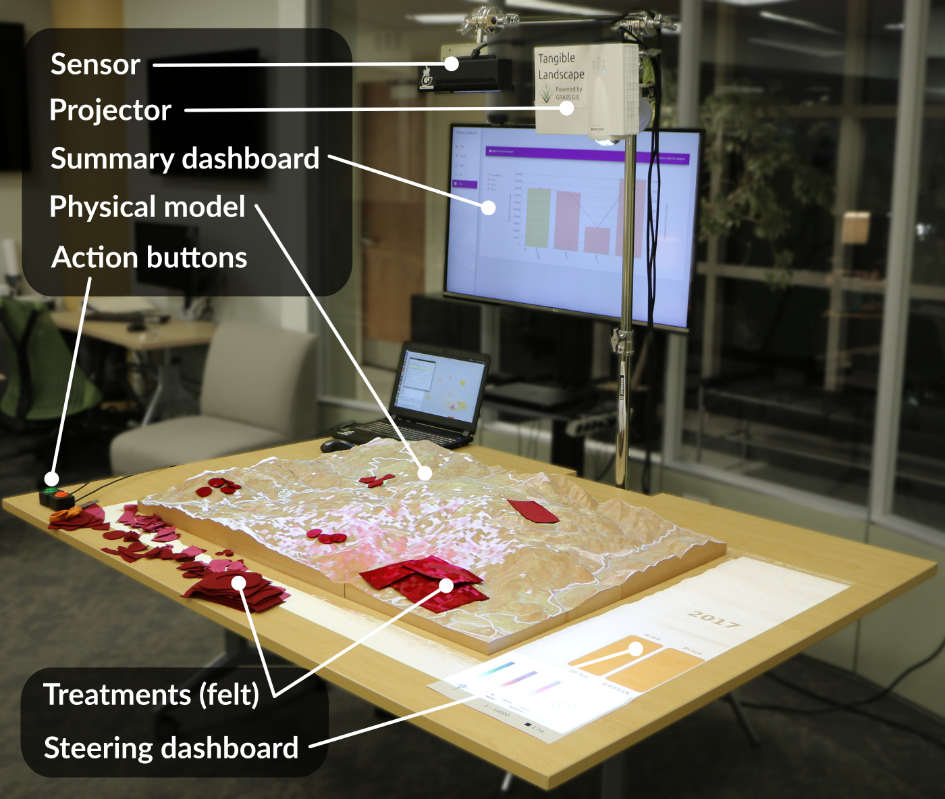


Figure B2. Example of Tangible Landscape setup for pathogen modelling. Credit: Anna Petrasova


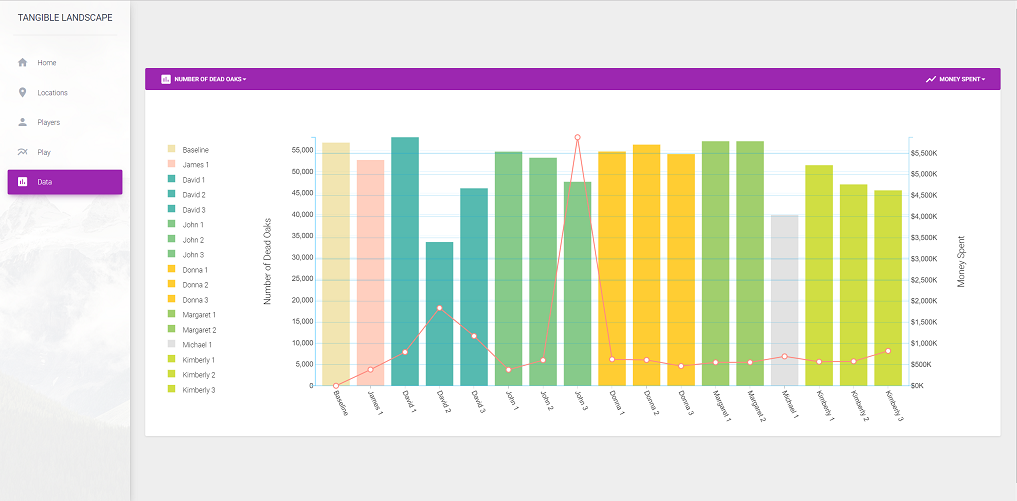


Figure B3: Example of web-based dashboard output. The bar graph and line graphs represent number of infected trees and money spent in each scenario, respectively. Bar colors represent which stakeholder designed each scenario. Credit: Makiko Shukunobe

REFERENCES

1  Tonini F, Shoemaker D, Petrasova A, Harmon B, Petras V, Cobb RC, Mitasova H, Meentemeyer RK. Tangible geospatial modeling for collaborative solutions to invasive species management. Environmental Modelling & Software. 2017 Jun 1;92:176-88. (doi: 10.1016/j.envsoft.2017.02.020)

2  Meentemeyer RK, Cunniffe NJ, Cook AR, Filipe JA, Hunter RD, Rizzo DM, Gilligan CA. Epidemiological modeling of invasion in heterogeneous landscapes: spread of sudden oak death in California (1990–2030). Ecosphere. 2011 Feb 1;2(2):1-24. (doi: 10.1890/es10-00192.1)

3  Cunniffe NJ, Cobb RC, Meentemeyer RK, Rizzo DM, Gilligan CA. Modeling when, where, and how to manage a forest epidemic, motivated by sudden oak death in California. Proceedings of the National Academy of Sciences. 2016 May 17;113(20):5640-5. (doi: 10.1073/pnas.1602153113)

4  Rizzo DM, Garbelotto M, Hansen EM. Phytophthora ramorum: integrative research and management of an emerging pathogen in California and Oregon forests. Annu. Rev. Phytopathol.. 2005 Jul 28;43:309-35. (doi: 10.1146/annurev.phyto.42.040803.140418)

5  Hansen EM, Kanaskie A, Prospero S, McWilliams M, Goheen EM, Osterbauer N, Reeser P, Sutton W. Epidemiology of Phytophthora ramorum in Oregon tanoak forests. Canadian Journal of Forest Research. 2008 Apr 24;38(5):1133-43. (doi: 10.1139/x07-217)

6  Peterson EK, Hansen EM, Kanaskie A. Temporal epidemiology of sudden oak death in Oregon. Phytopathology. 2015 Jul 6;105(7):937-46. (doi:10.1094/phyto-12-14-0348-fi)

7  Václavík T, Kanaskie A, Hansen EM, Ohmann JL, Meentemeyer RK. Predicting potential and actual distribution of sudden oak death in Oregon: prioritizing landscape contexts for early detection and eradication of disease outbreaks. Forest Ecology and Management. 2010 Aug 15;260(6):1026-35. (doi: 10.1016/j.foreco.2010.06.026)

8 Ohmann JL, Gregory MJ. Predictive mapping of forest composition and structure with direct gradient analysis and nearest-neighbor imputation in coastal Oregon, USA. Canadian Journal of Forest Research. 2002 Apr 1;32(4):725-41. (doi: 10.1139/x02-011)

9  PRISM Climate Group, Oregon State University, <http://prism.oregonstate.edu>, created 4 Feb 2004

10  GRASS Development Team, 2018. Geographic Resources Analysis Support System (GRASS) Software, Version 7.4. Open Source Geospatial Foundation. <http://grass.osgeo.org>

11  Petrasova A, Harmon B, Petras V, Tabrizian P, Mitasova H. Tangible modeling with open source GIS: Second Edition. New York: Springer International Publishing; 2018. (doi: 10.1007/978-3-329-89303-7)
