# Supplementary material for: Forecasting and control of emerging infectious forest disease through participatory modelling
Source: Philos Trans R Soc Lond B Biol Sci. 2019 May 20;374(1776):20180283. doi: 10.1098/rstb.2018.0283 (PMC6558554; doi:10.1098/rstb.2018.0283)
Supplement: Appendix C: Participatory Modelling Workshop [file rstb20180283supp3.docx]

Electronic Supplemental Material for “Forecasting and control of emerging infectious forest disease through participatory modelling”

Appendix C: Participatory Modelling Workshop:

*Description of Workshop:*

We held the first of a series of participatory modelling workshops with stakeholders at the Oregon Department of Forestry headquarters in Salem, Oregon in October, 2017. Twelve participants from Oregon State University (5), Oregon Department of Forestry (6), and the United States Forest Service (1) attended. The workshop lasted the entire day, and was divided into discrete sections. In the morning prior to interaction with the model, participants were asked to take a pre-workshop survey to assess their baseline knowledge of both the disease system and disease modelling tools. Then we held a group discussion focused on the implications of disease spread and the challenges of management. This discussion served two purposes: 1) to get participants thinking about the disease system, and 2) to give researchers valuable context on the participants’ disease concerns. We then familiarized participants with model functionality by giving a short presentation detailing all of the input data, input parameters, and model functions. To get everyone on a common page, participants were encouraged to ask clarifying questions and challenge aspects of the model or data they did not understand or did not agree with during this presentation and throughout the rest of the workshop.

We held two modelling sessions, each lasting a few hours. In the first, participants worked together to control disease spread starting with infection locations from 2016. Since there were less infections in 2016, it was easier for participants to learn model functions and explore different spatial management scenarios. In the second session, participants worked together to manage the 2017 infections, which were more challenging to control. After each of these sessions, there was a short debrief where we collectively examined the model outputs and discussed which scenarios worked best and why. After both modelling sessions, participants took a post-workshop survey designed to capture participant’s opinions of the system and any suggested updates. Following this workshop, we held one final group discussion to provide a forum for any final comments or feedback.

*Survey Results:*

All workshop participants completed pre- and post-workshop surveys designed to assess participants’ knowledge of forest and disease dynamics, perception of the model and Tangible Landscape, and to systematically collect recommended changes. Survey questions were a mix of rank-choice, open-ended, and Likert scales. We report a subset of these survey responses most related to credibility of model functions, interactivity and accessibility of the system, and potential applications in Fig. 3 in the main text. We further report here on familiarity with disease simulation models (Fig. C1), self-assessed learning (Table C1), and suggestions for model improvement (Table C2).


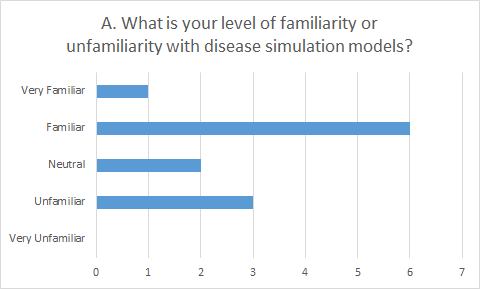


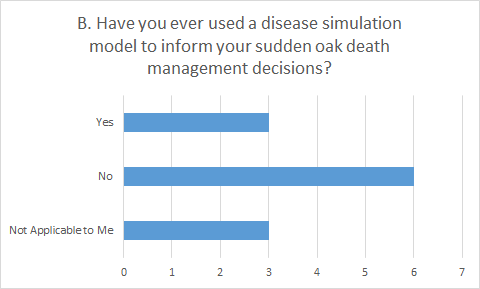


Figure C1: Survey results indicating participant’s familiarity (A1a) and prior experience with (A1b) disease simulation models.

| **Table C1: Things Learned from Workshop:** |
| --- |
| The potential effectiveness of alternative treatments (ie: host elimination). Unlikely to eradicate EU1 regardless of treatment if model predictions are accurate. |
| The many aspects of spread and treatment dynamics came out from the various participants. It was very interesting to have the breadth of knowledge in the room. I would like to have the system to “game” many different projects. |
| Reaffirmed belief that effective visual displays are helpful in learning. Even more effective is the self-learning gained by “gaming” the system. Would be extremely helpful with stakeholders. |
| The model is beginning to approach reality. Confirmed my suspicion that eradication is nearly impossible. |
| I learned about the model and getting to use tangible landscapes with respect to SOD. I also learned how important adequate funding was to disease management. |
| The model was interesting and the ability to compare treatment proposals would be very valuable. With a refinement in vegetation type, I think the model has a lot of potential for identifying areas at high risk for infection. |
| Management of SOD is going to be successful only if agencies, public, industry, and funding enables it to be so. The potential for eradication is small and may be unrealistic as a goal. But, confinement and treating new outbreaks could reduce the severity and landscape distribution of SOD. In short, SOD won’t go away but perhaps it can be managed. |
| Eradicating EU1 in Oregon is going to be almost impossible, even with budget surpassing 10 or 20 million dollars. |
| The simulations highlighted our uncertainty of detecting rare dispersal events - ie, at low frequencies are we accurately describing the distribution or must greater local intensification occur before detection. |
| How the tangible landscape model works and it’s possibilities. New way of displaying data (target). We are still uncertain about spread dynamics. Different ways we can use the model. |
| I learned the value of pre-emptive removal of tanoak in a high risk area. I learned the value of managing disease foci (isolated outbreaks). I learned the importance of strategic choice of management sites for cost effectiveness. Great workshop!!! What a wonderful tool to explore management strategies! |
| Better understanding of disease spread over time, especially in the absence of treatments. The epidemiological model needs to be adjusted to specifically reflect SOD spread in Oregon forests. The technology is fantastic as a tool for handling large amounts of information and displaying results effectively. |

Table C1: Participants’ open-end responses about what they learned during the workshop.

| **Table C2: Suggested Model Changes:** |
| --- |
| As was discussed, the need for more accurate, reliable and spatially fine scale inputs is a needed factor. These would help to refine the models and increase applicability. |
| Add adaptive management. Conduct model validation from real outbreaks. |
| I think the workshop would be great if the model was refined in terms of tanoak density, weather, and the ability to conduct treatments on a yearly basis. Great first workshop. |
| I think that it would be interesting to be able to add treatments on a yearly basis. |
| I think it might be interesting to have an example of a recalcitrant landowner. Either as a means to understand how a hole in treatment would or wouldn’t impact SOD control effectiveness. I also think it might be helpful to have some parameter variations (span a range of uncertainty) that might give a better idea of how robust or variable simulations might be for specific scenarios given uncertainty about phenomenological parameters (eg, reinfection rates for treatment areas). |
| It was a great opportunity to learn. Thank you for all your hard work. It would be interesting to adjust/validate the model to Oregon-specific data, in particular, or historical treatment data, refined climate/weather data and refined host distribution data. Then, a follow-up workshop or report of findings disseminated to Oregon to OR natural resource agencies. |
| Adjust length of LDD tail to match weather phenomenon of a given simulated year (allow to vary) (ex wetter spring = longer tail). Better host distribution. Allow for larger scale reduction in tanoak (ie tanoak thinning vs total removal) to either (a) decrease change of infection or (b) reduce local transmission rate. Allow adaptive management. Overall need for validation. Use to get at the question of the validity of the assumptions notably that sites remain infective and weather condition variation between years is negligible. Overall very cool and, importantly, fun. I love the idea of scorer. Consider online tool (maybe simplify). Conceptually it map be a leap for some people to translate the number of infected trees/ha to a risk map. |
| Use dataset from one of the expansions and treatment scenarios and compare model with reality. Use untreated site data on expansion and compare. |
| Validate model assumptions (for example, spores/tree) by comparing predicted rate of spread with actual rate of spread using Oregon epidemiological data. Add capability for adaptive management (the ability to manage each year, not just the first year). Superimpose a risk map on the landscape, and use finer scale vegetation map and climate/weather map to fine tune the risk map. This would really help to inform the management decisions. Make the tanoak distribution more obvious (finer scale, visually obvious). Some number of years after trees are initially infected, have them die and no longer produce spores. Allow for separately modelling NA1 and EU1 genotypes. |
| Improve the tanoak distribution layer. Improve vegetation layer by accounting for Douglas-fir plantations. Allow for treatments to occur during each year of the simulation, rather than only at the beginning. Add an optimization function to help determine where and how big to make treatments. |

Table C2: Participants’ open-ended suggestions for model improvement.
